# Knockdown of POSTN Inhibits Osteogenic Differentiation of Mesenchymal Stem Cells From Patients With Steroid-Induced Osteonecrosis

**DOI:** 10.3389/fcell.2020.606289

**Published:** 2020-12-21

**Authors:** Lizhi Han, Song Gong, Ruoyu Wang, Shaokai Liu, Bo Wang, Guo Chen, Tianlun Gong, Weihua Xu

**Affiliations:** ^1^Department of Orthopaedics, Union Hospital, Tongji Medical College, Huazhong University of Science and Technology, Wuhan, China; ^2^Department of Rehabilitation, Wuhan No.1 Hospital, Wuhan Hospital of Traditional Chinese and Western Medicine, Wuhan, China

**Keywords:** mesenchymal stem cells, steroid-induced osteonecrosis of femoral head, glucocorticoids, periostin, sclerostin, Wnt/β-catenin signaling pathway, osteogenic differentiation

## Abstract

Steroid-induced osteonecrosis of femoral head (SONFH) is a common and serious complication caused by long-term and/or excessive use of glucocorticoids (GCs). The decreased activity and abnormal differentiation of bone marrow mesenchymal stem cells (BMSCs) are considered to be one of the major reasons for the onset and progression of this disease. Periostin (POSTN) is a matricellular protein which plays an important role in regulating osteoblast function and bone formation. Sclerostin (SOST) is a secreted antagonist of Wnt signaling that is mainly expressed in osteocytes to inhibit bone formation. However, the exact role of POSTN and SOST in SONFH has not been reported yet. Therefore, we detected the differential expression of POSTN and SOST in BMSCs of SONFH Group patients, and Control Group was patients with traumatic ONFH (TONFH) and developmental dysplasia of the hip (DDH). Furthermore, we used lentiviral transfection to knockdown POSTN expression in BMSCs of patients with SONFH to study the effect of POSTN knockdown on the SOST expression and osteogenic differentiation of BMSCs. The results indicated that the endogenous expression of POSTN and SOST in BMSCs of SONFH Group was upregulated, compared with Control Group. POSTN was upregulated gradually while SOST was downregulated gradually at days 0, 3, and 7 of osteogenic differentiation of BMSCs in Control Group. Contrarily, POSTN was gradually downregulated while SOST was gradually upregulated during osteogenic differentiation of BMSCs in SONFH Group. This could be due to increased expression of SOST in BMSCs, which was caused by excessive GCs. In turn, the increased expression of POSTN in BMSCs may play a role in antagonizing the continuous rising of SOST during the osteogenic differentiation of BMSCs in patients with SONFH. POSTN knockdown significantly attenuated osteo-specific gene expression, alkaline phosphatase activity, and calcium nodule formation *in vitro*; thus inhibiting the osteogenic differentiation of BMSCs in patients with SONFH. Besides, POSTN knockdown upregulated SOST expression, increased GSK-3β activity, and downregulated β-catenin. These findings suggest that POSTN have an essential role in regulating the expression of SOST and osteogenic differentiation of BMSCs in patients with SONFH, and POSTN knockdown suppresses osteogenic differentiation by upregulating SOST and partially inactivating Wnt/β-catenin signaling pathway. Therefore, targeting POSTN and SOST may serve as a promising therapeutic target for the prevention and treatment of SONFH.

## Introduction

Osteonecrosis of the femoral head (ONFH) is reported to be a refractory skeletal disorder bothering over 20 millions of people across the world, and presenting an increasing trend (Chen et al., 2019). ONFH can be categorized as traumatic and non-traumatic factors according to its etiologies, mainly affecting young and active adults in the third to fifth decades of life (Mont and Hungerford, 1995). If not treated timely, the natural history of this disease frequently includes the fracture of the subchondral bone, progressive collapse of the femoral head, and degenerative arthritis with substantial pain and dysfunction of the hip (Mont et al., 2006c). Trauma leading to fracture of the femoral neck or dislocation of the hip is the major cause of traumatic ONFH (TONFH). High-dose glucocorticoids (GCs) and long-term alcohol abuse are well-known risk factors to non-traumatic ONFH (Larson et al., 2018). Excessive use of GCs has become one of the most common etiological factors for non-traumatic ONFH because of their anti-inflammatory effects to treat chronic inflammatory diseases such as asthma, arthritis, allergic shock, and inflammatory bowel disease (Mont et al., 2006a; Hartmann et al., 2016). Especially the widespread clinical application of GCs used as adjuvant therapy for epidemic viral pneumonia during outbreaks of coronavirus disease 2019 (COVID-19) (Yang et al., 2020), steroid-induced osteonecrosis of femoral head (SONFH) may further increase in the future. Currently, the exact pathogenesis of SONFH is still unclear, although it is considered to be the result of the combined action of multiple mechanisms, such as the lipid metabolism disorder theory, insufficient blood supply theory, the inflammation and cell apoptosis theory, and so forth (Wang et al., 2018). Among them, cell apoptosis and dysfunction of bone marrow mesenchymal stem cells (BMSCs) are considered to be involved in the onset and progression of this disease (Han et al., 2019).

Mesenchymal stem cells (MSCs), a population of non-hematopoietic stem cells initially isolated from bone marrow, are capable of self-renewal with multilineage differentiation potential to differentiate into several mesenchymal tissues, including bone, cartilage, and fat (da Silva Meirelles et al., 2006). When cultured *in vitro*, these cells exhibit a stable phenotype and maintain as a monolayer, and can be induced to differentiate into osteoblasts, adipocytes, or chondrocytes under different culture conditions (Pittenger et al., 1999). Besides, BMSCs are easily isolated and obtained without immunogenicity and infectiousness that make them ideal candidates for the applications in tissue engineering and regenerative medicine (MacFarlane et al., 2013). Physiological concentrations of GCs promote osteogenic differentiation of MSCs and bone formation, however, high doses of GCs suppress osteoblast differentiation and bone formation, and shifts the differentiation commitment of MSCs from the osteoblastic lineage to the adipocyte lineage (Han et al., 2019). In addition to inhibiting osteoblast differentiation of osteoprogenitor cells, high doses of GCs can also induce apoptosis of osteoblasts and osteocytes and reduce their proliferation (Hartmann et al., 2016). Besides, with excessive use of GCs including high doses or a long course of treatment, the incidence of GC’s skeletal side effects including osteonecrosis, osteoporosis and fractures were also increased accordingly (Weinstein, 2012). The intrinsic deficiency in osteogenic differentiation found in BMSCs isolated from patients with SONFH potentially suggests that an insufficient repair mechanism wound cause osteonecrosis (Houdek et al., 2016).

Periostin (POSTN), originally known as osteoblast-specific factor 2, is a highly conserved extracellular matrix protein and widely expressed in the body including the skeleton. It is mainly produced during ontogenesis and in adult connective tissues submitted to mechanical loading, such as bone, tendon, periodontal ligament, heart valves, skin (Conway et al., 2014; Bonnet et al., 2016). In bone, POSTN acts as a structural molecule of bone matrix regulating collagen cross-linking, and as a signaling molecule interacting with integrin receptors and Wnt/β-catenin pathways to promote osteoblast functions and bone formation (Bonnet et al., 2016). POSTN may play an important role in promoting periosteal callus formation and repair of bone fractures during the early stage of fracture healing (Nakazawa et al., 2004). Besides, POSTN knockout mice (POSTN*^–/–^*) have exhibited osteoporosis with decreased bone mineral density (BMD), deteriorated bone microarchitecture and low bone strength (Gerbaix et al., 2015). Sclerostin (SOST) was discovered during the study of sclerosteosis (Balemans et al., 2001) and Van Buchem disease (Balemans et al., 2002). SOST is a glycoprotein mainly expressed in osteocytes and inhibits bone formation by antagonizing the Wnt/β-catenin signaling pathway (ten Dijke et al., 2008). Romosozumab, one of the monoclonal antibodies against SOST, can reduce the inhibition of Wnt signaling and improve osteoblast function (Reid, 2017).

Wnt signaling pathway is essential for proliferation, renewal, and differentiation of stem cells during embryonic development and adult tissue homeostasis, including the differentiation of MSCs into osteoprogenitor cells and chondrocytes (Logan and Nusse, 2004; Houschyar et al., 2018). Wnt/β-catenin pathway, also termed the canonical Wnt pathway, is activated upon the binding of Wnt ligands to a dual receptor complex comprising low-density lipoprotein receptor-related protein 5 or 6 (LRP5/6) co-receptors and the frizzled receptor at the cell membrane (Baron and Kneissel, 2013), causing the suppression of glycogen synthase kinase-3β (GSK3β) activity and preventing phosphorylation of β-catenin and its proteosomal degradation (MacDonald and He, 2012). Upon activation of Wnt, β-catenin accumulates in the cytoplasm and translocates into the nucleus, where it promotes T-cell factor/lymphoid enhancing factor (TCF/LEF) mediated transcription while controlling target gene transcription (Molenaar et al., 1996; Eastman and Grosschedl, 1999). High doses of GCs promote the expression of antagonists of Wnt signaling pathway, such as dickkopf-1 (DKK1) (Ohnaka et al., 2005; Butler et al., 2010) and SOST (Yao et al., 2008; Adhikary et al., 2019). Conversely, glucocorticoid-induced osteopenia in rats is rescued by knocking down DKK1 (Wang et al., 2008).

Although it has been well documented that POSTN and SOST play an important role in regulating bone formation and remodeling especially in bone’s adaptive response to mechanical loading (Nakazawa et al., 2004; van Bezooijen et al., 2004; Bonnet et al., 2009; Gerbaix et al., 2015), the endogenous expression levels of POSTN and SOST in human BMSCs (hBMSCs) of patients with SONFH and their effects on osteogenic differentiation remain to be clarified. Besides, whether there is a specific relationship between the expression of POSTN and SOST during osteogenic differentiation of hBMSCs in patients with SONFH, and whether they affect osteogenic differentiation via Wnt/β-catenin signaling, also need to be further investigated. In this study, we determined the endogenous expression of POSTN and SOST, and found that the expression levels of both POSTN and SOST were increased significantly in hBMSCs of SONFH Group, compared with Control Group. Furthermore, we found that the expression of POSTN was increased gradually while SOST was reduced gradually at days 0, 3, and 7 of osteogenic differentiation of hBMSCs in Control Group. In contrast, the expression of POSTN was gradually decreased while SOST was gradually increased during osteogenic differentiation of hBMSCs in SONFH Group. We hypothesized that downregulation of POSTN upregulates SOST expression and attenuates osteogenesis of hBMSCs via Wnt/β-catenin signaling. By evaluating the expression levels of SOST and specific osteogenic markers and calcium deposition, we revealed that POSTN knockdown upregulates the expression of SOST and inhibits osteogenic differentiation of hBMSCs partly via the Wnt/β-catenin signaling pathway *in vitro*. Therefore, our study provides a promising stem cell-based strategy for tissue engineering and regenerative medicine including bone regeneration.

## Materials and Methods

### Source and Grouping of hBMSCs Samples

After institutional ethics committee approval, the bone marrow samples of all the included patients were obtained during operation. These patients underwent total hip arthroplasty (THA) from December 2018 to October 2019 at the authors’ institution (Wuhan Union Hospital, Huazhong University of Science and Technology). Eleven patients with SONFH and 12 patients (including three patients with developmental dysplasia of the hip and nine patients with TONFH) were assigned accordingly to SONFH Group and Control Group (Table 1). The study was approved by the Ethics Committee at Tongji Medical College, Huazhong University of Science and Technology, and written informed consent was signed from all subjects. The diagnosis for ONFH was confirmed by radiography and magnetic resonance imaging while developmental dysplasia of the hip (DDH) was confirmed by radiography. The inclusion criteria of each chosen individuals with ONFH were as follows: Association Research Circulation Osseous (ARCO) Stage IV to VI ONFH (Mont et al., 2006b) or DDH with advanced osteoarthritis, requiring THA for treatment, age from 20 to 60, without smoking or alcohol history. For patients with SONFH, the steroid exposure threshold was 1800 mg of prednisolone or its equivalent over 4 weeks (Koo et al., 2002), and there was a history of previous fracture of the femoral neck for patients included in TONFH Group. Besides, there was no corticosteroid use history for DDH and TONFH patients. Patients with infectious disease, immunodeficiency, hyperlipemia, poorly controlled diabetes (hemoglobin A1C > 8%), cardiopathy, hematological diseases, and malignant tumors were excluded.

**TABLE 1:** Comparison between patients in SONFH Group and Control Group.

Demographics	SONFH Group (*n* = 11)	Control Group (*n* = 12)	*P*-value
Males	6	6	1.0
Females	5	6	
Age (years)	38.2 ± 3.4	45.3 ± 3.6	0.1693
BMI (kg/m^2^)	26.06 ± 0.57	25.76 ± 0.42	0.6750

### Isolation and Culture of hBMSCs

Bone marrow aspirate (10–20 mL) was harvested from the proximal end of femur while inserting the reamer and tapering awl into the femoral canal during THA. The 10 ml bone marrow samples were diluted with 10ml phosphate-buffered saline (PBS; HyClone, United States) and added over 10 ml Percoll solution (Sigma, United States) with a density of 1.073 g/ml in a 50 mL conical tube. Mononuclear cells from the bone marrow were isolated by density gradient centrifugation (472 *g* for 30 min). Then, cells were harvested and resuspended in complete culture medium containing low-glucose complete Dulbecco’s modified Eagle’s medium (L-DMEM; HyClone, United States), 10% fetal bovine serum (FBS; Gibco, United States), and 1% penicillin-streptomycin (Invitrogen). These cells were seeded at a density of 5000 cells/cm^2^ in 25-cm^2^ culture flasks (Corning, United States) and incubated at 37°C in a humidified atmosphere containing 5% carbon dioxide. After 1 day interval, the non-adherent cells were removed and fresh cultures were added every 3 days. When cells reached ≥ 80% confluence, they were digested using 0.25% trypsin/EDTA (HyClone, United States) and replated at a 1:2 dilution for initial subculture. The cells were expanded by successive subculture *in vitro*, and cells from the second or third passage were ready for the following experiments.

### Identification of hBMSCs

At the third passage, the cell samples in SONFH Group and Control Group were respectively performed the immunophenotype analysis to identify human MSC phenotypes using flow cytometry. For each sample, about 10^6^ cells were incubated for 30 min at 4°C with the following fluorescein isothiocyanate (FITC) or phycoerythrin (PE) conjugated anti-human primary antibodies, such as CD34-PE, CD105-PE, HLA-DR-PE, CD73-FITC, and CD90-PE (all purchased from BioLegend, United States). Negative control (no antibody treatment) was included for comparison. Cells were washed twice and resuspended in PBS, and data were acquired on a CytoFLEX (Beckman Coulter, United States) flow cytometer. MSCs were characterized by the expression of CD73, CD90, and CD105 and the lack of HLA-DR and CD34 expression. In addition, after plating bone marrow-derived MSCs to a 6-well culture plate with a density of 5000 cells/cm^2^, the cells were grown to confluency. Then, the trilineage differentiation potential of MSCs was determined in osteogenic, adipogenic, and chondrogenic differentiation mediums respectively (Cyagen, China). After cells were differentiated into osteoblasts, adipocytes, and chondrocytes in respective induction mediums, Alizarin red S (ARS) staining, Oil Red O staining, and Alcian blue staining were performed to confirm each lineage differentiation respectively according to manufacturer protocols.

### Lentiviral Packaging and Cell Infection

Cell samples of three different patients were randomly selected from the sample pool of SONFH Group for lentivirus transfection. Lentiviral knockdown POSTN (lenti-POSTN) particles and lentiviral green fluorescent protein (GFP) particles, used as the control group (lenti-control), were prepared by Huameng Biotechnology Co., Ltd (Wuhan, China). For infections, 50–60% confluent primary hBMSCs at passages 2–3 were incubated with polybrene (5 μg/mL) and lentiviral particles in L-DMEM medium at a multiplicity of infection (MOI) of 50. After incubation for 12 h, more than 95% of the cells were still viable, and then the culture medium was replaced with fresh medium. After 72 h, all transfected cells were passaged and used for subsequent experiments. The mRNA and protein levels of POSTN were determined by quantitative real-time PCR (qPCR) and western blotting analyses.

### Cell Viability Assay

To evaluate the effect of POSTN knockdown on the proliferation of hBMSCs, cells were seeded into a 96-well plate at 5000 cells/well and allowed to adhere for 24 h. After 24 h, the medium was replaced by 100 μl fresh L-DMEM (without FBS) medium containing 10% Cell Counting Kit-8 (CCK-8; Beyotime, China) and incubated at 37 °C for 2 h. The absorbance was measured at 450 nm using a microplate reader (BioTek, United States) and was in direct proportion to cell proliferation.

### Osteogenic Differentiation Protocol

Briefly, hBMSCs were plated at a density of 2 × 10^4^/cm^2^ on 6-well cell culture plates and cultured with L-DMEM medium containing 10% FBS and 1% penicillin-streptomycin at 37°C under 5% CO_2_ for 48 h. When the cells reached 60–70% confluence, the culture medium was replaced with osteogenic induction medium (L-DMEM supplemented with 10% FBS, 1% penicillin-streptomycin, 0.2 mM ascorbic acid, 100 nM dexamethasone, and 10 mM β-glycerophosphate). The osteogenic induction medium was maintained and refreshed every 2–3 days by adding fresh medium.

### Alkaline Phosphatase Staining and Alkaline Phosphatase Activity Assay

After osteogenic induction of hBMSCs for 7 days, cells were rinsed twice with PBS and fixed with 4% paraformaldehyde for 15 min, then washed twice with PBS again and stained using the BCIP/NBT Alkaline Phosphatase (ALP) Color Development Kit (Beyotime, Nanjing, China) according to the manufacturer’s instructions. To measure the ALP activity, cells were lysed using lysis buffer (20 mM Tris/HCl [pH 7.5], 150 mM NaCl, 1% Triton X-100). ALP activity was tested using the ALP Activity Assay Kit (Beyotime) as described in the manufacturer’s protocols. Briefly, after hBMSCs were cultured in osteogenic induction medium for 7 days, the conversion of colorless *p*-nitrophenyl phosphate to colored *p*-nitrophenol was detected at the wavelength of 405 nm.

### Alizarin Red Staining

After osteogenic induction of hBMSCs for 9 or 14 days, ARS staining was performed to assess mineral deposition. Cells were washed twice with PBS and fixed with 4% paraformaldehyde for 20 min at room temperature. Then the cells were rinsed three times with distilled water and subsequently stained with 0.5% Alizarin red working solution (Cyagen Biosciences, China) for 30 min at room temperature, followed by washing with distilled water again. The effect of cell staining was observed and photos were taken under a light microscope. For quantification of the mineralized area, the stained monolayer was destained by incubating with 10% cetylpyridinium chloride (Sigma, China) at room temperature for 1 h. Then 200 μl of the collected solution was added into 96-well plates and read at the wavelength of 570 nm using a microplate reader (BioTek, United States). The readings were used to normalize the results as the total protein concentration.

### Oil Red O Staining

After adipogenic induction of hBMSCs for 3 weeks, cells were stained with Oil Red O solution. The working solution was prepared by diluting the stock solution (0.5% Oil Red O in isopropanol) with distilled water (3:2), and then the solution was filtered. After rinsing twice with PBS, cells were fixed with 4% paraformaldehyde for 30 min at room temperature. After fixation, the cells were washed twice with distilled water and stained using Oil Red O solution for 30 min at room temperature. To remove the unbound excessive dye, the stained cells were washed three times with distilled water, then observed and photographed under a light microscope. To quantify the intracellular lipid accumulation, 100% isopropanol was added to each well to extract the incorporated dye. The extracted dye was then transferred to a 96-well plate, after which the absorbance was measured using a spectrophotometer at 450 nm.

### RNA Extraction and qPCR

Total cellular RNA was extracted using Trizol reagent (TAKARA, Japan) and then reverse-transcribed into cDNA with PrimeScript^TM^ RT Master Mix (TAKARA, Dalian, China) according to the manufacturer’s instructions. The messenger RNA (mRNA) expressions of target genes were quantified by qRT-PCR, using the One Step SYBR^®^ PrimeScript^TM^ RT-PCR Kit (TAKARA). The CT values were normalized to glyceraldehyde 3-phosphate dehydrogenase (GAPDH) expression and relative target gene expression levels were analyzed using the 2^–ΔΔCt^ method. The primer sequences used in present study are listed in Table 2.

**TABLE 2:** The primer sequences used for quantitative real-time PCR.

Gene	Forward primer sequence (5′→3′)	Reverse primer sequence (5′→3′)
POSTN	CACTCTTTGCTCCCACCAAT	TCAAAGACTGCTCCTCCCATA
SOST	GGAATGATGCCACAGAGGTCAT	CCCGGTTCATGGTCTGGTT
RUNX2	ACTTCCTGTGCTCGGTGCT	GACGGTTATGGTCAAGGTGAA
COL1A1	GAGAGCATGACCGATGGATT	CCTTCTTGAGGTTGCCAGTC
OSX	CCTGCGACTGCCCTAATT	GCGAAGCCTTGCCATACA
OPN	ATGATGGCCGAGGTGATAGT	ACCATTCAACTCCTCGCTTT
β-catenin	TTAAGCCTCTCGGTCTGTGG	GCCGCTTTTCTGTCTGGTTC
Axin	TTATGCTTTGCACTACGTCCCT CCA	CGCAACATGGTCAACCCTCA GAC
GSK3β	GACTAAGGTCTTCCGACCCC	TTAGCATCTGACGCTGCTGT
GAPDH	GAAAGCCTGCCGGTGACTAA	TGGAATTTGCCATGGGTGGA

### Western Blotting Analysis

Cells were lysed and the total cell protein was extracted in RIPA buffer containing a proteasome inhibitor (Beyotime, China). Protein concentrations were measured using a BCA protein assay kit (Beyotime). Equal protein amounts were separated on a 10% sodium dodecyl sulfate-polyacrylamide gel electrophoresis (SDS-PAGE) and then transferred onto a polyvinylidene fluoride (PVDF) membrane (Millipore, Shanghai, China). After blocking in 5% defatted milk for 2 h, the membranes were incubated with specific primary antibodies overnight at 4°C. Primary antibodies used in the present study include anti-GAPDH (1:10000, Proteintech, United States), anti-POSTN (1 μg/ml; Abcam, China), anti-SOST (1 μg/ml; Abcam), anti- RUNX2 (1 μg/ml; Abcam), anti-COL1A1 (1: 1000; Abcam), anti-Active (non-phosphorylated) β-catenin (1: 1000; Cell Signaling Technology), and anti-Total β-catenin (1: 1000; Cell Signaling Technology). After washing with TBST for four times, the membranes were incubated with horseradish peroxidase (HRP)-conjugated secondary antibodies (Sigma, United States) for 1 h at room temperature. After washing with TBST for five times, the PVDF membranes were analyzed using an enhanced chemiluminescent detection reagent (Millipore) and scanned with the BioSpectrum Imaging System (UVP, Germany).

### Statistical Analysis

Statistical analysis was conducted using the GraphPad Prism 8.0 (GraphPad Software, CA, United States). All data were presented as mean ± standard deviation (SD). Comparison between two groups was carried out by a two-tailed Student’s *t*-test, and comparisons between multiple groups were performed using one-way analysis of variance (ANOVA) followed by Tukey’s test or two-way ANOVA followed by Bonferroni’s *post hoc* test. *P*-values < 0.05 were considered to indicate statistically significant differences.

## Results

### Identification and Growth Morphology of hBMSCs

**FIGURE 1:**
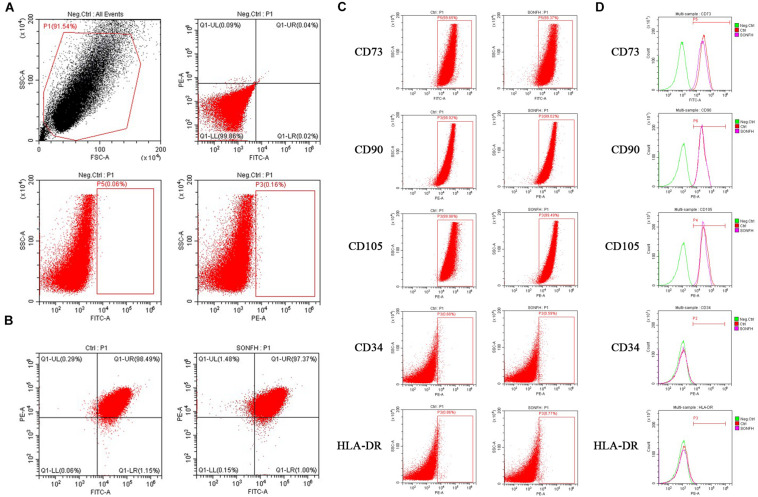
Flow cytometry analyses of hBMSCs from SONFH Group and Control Group. **(A)** Representative flow cytometry plots of hBMSCs without fluorescence antibodies (negative control). **(B)** Expression of hBMSCs surface markers CD90-PE, CD73-FITC (left panel for Control Group; right panel for SONFH Group). **(C)** Representative flow cytometry dot-plots showing high expression levels of CD73, CD90, and CD105 and low expression levels of CD34 and HLA-DR on the surface of hBMSCs (left panel for Control Group; right panel for SONFH Group). **(D)** Representative flow cytometry histograms of hBMSCs labeled with antibodies against CD73, CD90, CD105, CD34, and HLA-DR, respectively or without fluorescence antibodies (negative control) in Control Group and SONFH Group. FSC, forward scatter; SSC, side scatter. Neg.Ctrl refers to negative control; Ctrl and SONFH refer to Control Group and SONFH Group, respectively.

**FIGURE 2:**
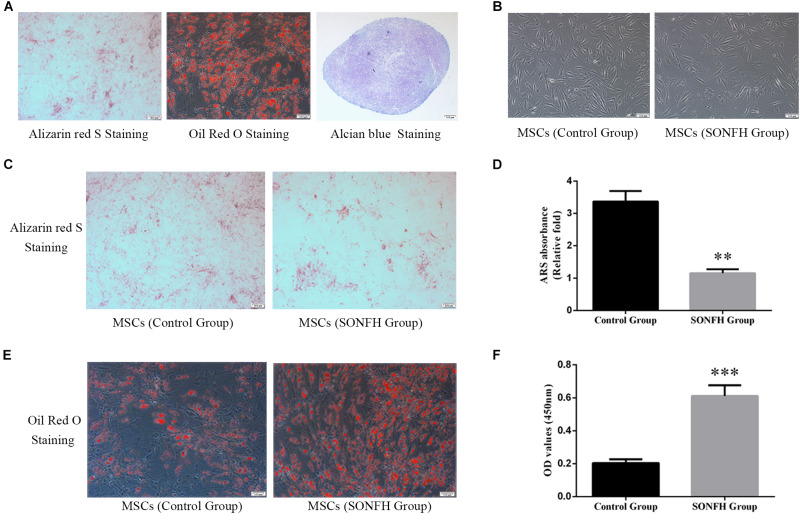
Tri-lineage differentiation of hBMSCs, and the comparison of osteoblast differentiation and adipogenesis differentiation capacity between hBMSCs of SONFH Group (*n* = 11) and Control Group (*n* = 12). **(A)** The tri-lineage differentiation capacity of hBMSCs to differentiate into the osteogenic, adipogenic, and chondrogenic lineages was determined by ARS staining (left panel, scale bar: 200 μm), Oil Red O staining (middle panel, scale bar: 100 μm) and Alcian blue staining (right panel, scale bar: 100 μm), respectively. **(B)** Representative images taken by inverted microscope displayed the growth morphology of the primary hBMSCs in SONFH Group and Control Group on the 9th day. Slower cell reproduction and higher morphological variation were observed in hBMSCs of SONFH Group (right panel, scale bar: 100 μm) compared with Control Group (left panel, scale bar: 100 μm). **(C)** Representative images of ARS staining in SONFH Group and Control Group after hBMSCs treated with osteoblast differentiation medium for 9 days (scale bar: 200 μm). **(D)** Calcium deposits for matrix mineralization were quantified by the extraction of ARS-positive deposits. The quantitative analysis showed significant statistical differences between SONFH Group and Control Group. **(E)** Representative images of Oil Red O staining in SONFH Group and Control Group after hBMSCs treated with adipogenic differentiation medium for 21 days (scale bar: 100 μm). **(F)** Quantitative analysis of Oil Red O staining between SONFH Group and Control Group. ^∗∗^*P* < 0.01 versus Control Group, ^∗∗∗^*P* < 0.001 versus Control Group.

In order to prove that the cells isolated and cultured *in vitro* are MSCs, we identified five surface antigens of human MSCs different from hemocytes and other monocytes by flow cytometry analysis. Flow cytometry analysis showed that more than 98% of the isolated third-passage cells in both SONFH Group and Control Group were positive for the surface markers CD73, CD90, and CD105, and negative for CD34 and HLA-DR (Figures 1A–D). Multilineage differentiation capacity of MSCs toward osteoblasts, adipocytes, and chondrocytes in respective induction mediums was demonstrated by ARS staining, Oil Red O staining, and Alcian blue staining respectively (Figure 2A). These results indicated that the cells cultured *in vitro* are human MSCs, which meet the criteria of the International Society for Cell Therapy (ISCT) to define MSCs (Dominici et al., 2006). After primary culture for 7–10 days, hBMSCs were identified with a spindle-shaped and flat appearance (Figure 2B). Bone marrow mesenchymal stem cells (BMSCs) isolated from patients of SONFH Group showed slower cell reproduction and higher morphological variation when compared with Control Group (Figure 2B).

### The Attenuated Osteogenic Differentiation and Enhanced Adipogenic Differentiation of hBMSCs in SONFH Group When Compared With Control Group

The osteogenic and adipogenic differentiation induction of hBMSCs in SONFH Group and Control Group were stained with ARS staining and Oil Red O staining respectively. The difference of coloration between SONFH Group and Control Group after ARS staining and Oil Red O staining was distinct (Figures 2C,E), and the quantitative analysis showed significant statistical differences between the two groups (Figures 2D,F), which indicated different potentials for osteogenic and adipogenic differentiation in the two groups of hBMSCs. The results of the stainings showed that the osteogenic differentiation capacity of hBMSCs in SONFH Group was attenuated, while the adipogenic differentiation capacity was enhanced when compared with Control Group.

### Elevated Endogenous POSTN and SOST Expression of hBMSCs in Patients With SONFH

**FIGURE 3:**
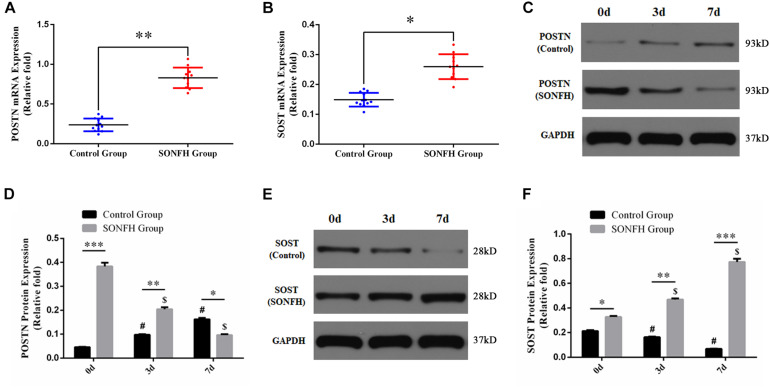
Endogenous POSTN and SOST expression of hBMSCs from all donors included in SONFH Group (*n* = 11) and Control Group (*n* = 12). **(A,B)** POSTN and SOST mRNA expression levels were determined by qPCR. **(C–F)** POSTN and SOST protein expression levels were determined by western blotting analysis at days 0, 3, and 7 of osteogenic differentiation. The relative mRNA and protein expression levels were normalized to the levels of glyceraldehyde-3-phosphate dehydrogenase (GAPDH). All experimental data are given as mean values ± SD. ^∗^*P* < 0.05 versus Control Group, ^∗∗^*P* < 0.01 versus Control Group, ^∗∗∗^*P* < 0.001 versus Control Group. ^#^*P* < 0.05 versus Control Group at day 0 or at day 7 of osteogenic differentiation. ^$^*P* < 0.05 versus SONFH Group at day 0 or at day 7 of osteogenic differentiation.

To determine the expression levels of POSTN and SOST in hBMSCs of patients with SONFH, we detected the mRNA and protein expression of POSTN and SOST in hBMSCs of all patients included in SONFH Group and Control Group. Compared with Control Group, the mRNA expression levels of POSTN and SOST in hBMSCs of SONFH Group were increased significantly (Figures 3A,B). Similarly, the protein expression levels of POSTN and SOST in SONFH Group before osteogenic differentiation of hBMSCs (designated as day 0 of osteogenic differentiation) were also increased significantly (Figures 3C–F). Besides, to analyze the expression levels of POSTN and SOST associated with osteogenic differentiation of hBMSCs, we examined the endogenous POSTN and SOST expression in hBMSCs of SONFH Group and Control Group at days 0, 3, and 7 during osteogenic differentiation. Compared with undifferentiated hBMSCs, the protein expression of POSTN in Control Group was increased gradually while SOST was decreased gradually at days 3 and 7 (Figures 3C–F). In contrast, the protein expression of POSTN was gradually reduced while SOST was gradually upregulated during the osteogenic differentiation of hBMSCs in SONFH Group (Figures 3C–F). Furthermore, the protein expression levels of POSTN in hBMSCs of SONFH Group were significantly lower than that of Control Group at day 7 of osteogenic differentiation (Figures 3C,D).

### POSTN Knockdown of hBMSCs in Patients With SONFH

**FIGURE 4:**
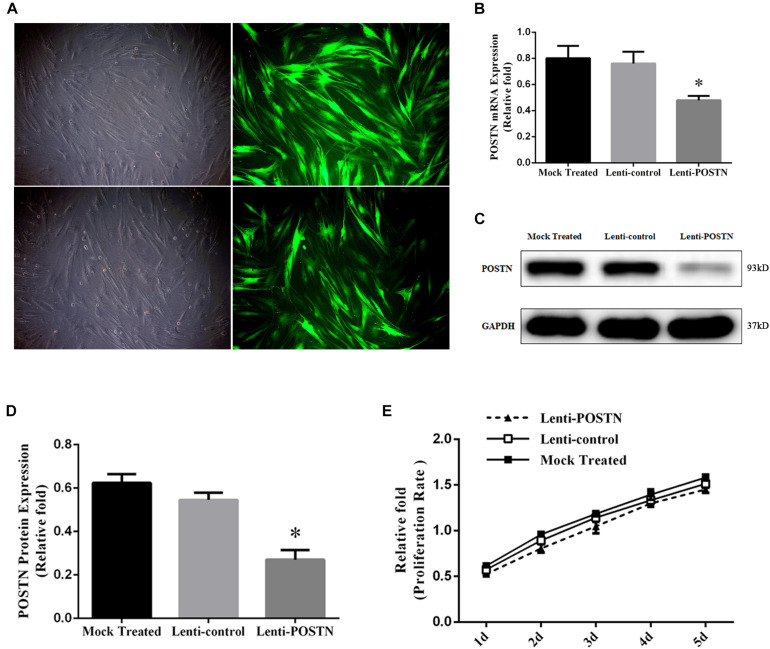
Construction of POSTN-konckdown hBMSCs and lenti-control hBMSCs and the impact of POSTN knockdown on the proliferation of hBMSCs from SONFH patients. **(A)** After lentiviral transfection and puromycin selection, hBMSCs in patients with SONFH were observed under an optical microscope and a fluorescence microscope (scale bar: 100 μm). **(B–D)** The expression levels of POSTN mRNA and protein were measured, respectively, by qPCR and western blotting analysis among the mock-treated, lenti-control, and lenti-POSTN group. **(E)** No significant difference in the cell proliferation rate of hBMSCs among mock-treated, lenti-control, and lenti-POSTN group indicated that POSTN knockdown did not affect the proliferation of hBMSCs in patients with SONFH. All experimental data are given as mean values ± SD and were confirmed by three independent experiments (*n* = 3) with cells from 3 different donors. ^∗^*P* < 0.05 versus the lenti-control group.

To elucidate the potential effect of POSTN on osteogenic differentiation of hBMSCs in patients with SONFH, we used a lentiviral vector system to efficiently knockdown the expression of POSTN in third-generation hBMSCs of three random patients from SONFH Group. Quantification of POSTN knockdown was assessed by the ratio of green fluorescent protein (GFP)-positive cells relative to the total cell number (Figure 4A). The expression levels of POSTN were quantified by qPCR and western blotting 72 h after infection and screening. Compared with the mock-treated (without virus) and lenti-control groups, the expression levels of POSTN were decreased significantly in the lenti-POSTN group (Figures 4B–D).

### POSTN Knockdown Did Not Affect the Proliferation of hBMSCs in Patients With SONFH

To determine whether the knockdown of POSTN affects the proliferation of hBMSCs in patients with SONFH, cell proliferation was measured with a CCK-8 assay. The effects of POSTN knockdown on the proliferation of hBMSCs at days 1, 2, 3, 4, and 5 post-infection are shown in Figure 4E. No significant difference in the cell proliferation rate of hBMSCs was identified among mock-treated, lenti-control, and lenti-POSTN group.

### POSTN Knockdown Decreased the Expression of Osteo-Specific Markers

**FIGURE 5:**
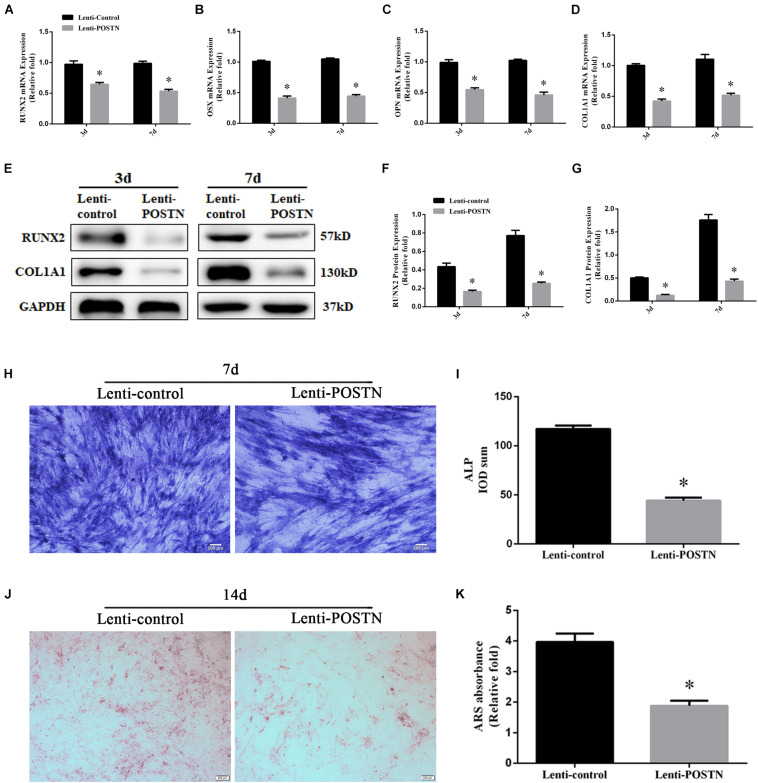
The knockdown of POSTN suppressed the osteogenic differentiation of hBMSCs in patients with SONFH. **(A)** The mRNA levels of RUNX2 were detected by qPCR on days 3 and 7 after osteogenic induction of hBMSCs. **(B)** The mRNA levels of OSX. **(C)** The mRNA levels of OPN. **(D)** The mRNA levels of COL1A1. **(E–G)** The protein levels of RUNX2 and COL1A1 were detected by western blotting analysis on days 3 and 7 after osteogenic induction of hBMSCs. **(H)** ALP staining was performed after the osteogenic differentiation of hBMSCs for 7 days (scale bar: 200 μm). **(I)** The ALP activity of hBMSCs after the osteogenic differentiation for 7 days. **(J)** ARS staining was performed after the osteogenic differentiation of hBMSCs for 14 days (scale bar: 200 μm). **(K)** Calcium deposits for matrix mineralization were quantified by the extraction of ARS-positive deposits. All experimental data are given as mean values ± SD and were confirmed by three independent experiments (*n* = 3) with cells from three different donors. ^∗^*P* < 0.05 versus the lenti-control group.

To assess the effects of POSTN knockdown on osteogenic differentiation of hBMSCs in patients with SONFH, the expression levels of osteo-specific markers, including runt-related transcription factor 2 (RUNX2), osterix (OSX), collagen type I alpha 1 chain (COL1A1), and osteopontin (OPN), were detected by qPCR and western blotting. The results of the qPCR analysis showed that the mRNA expression levels of RUNX2, OSX, COL1A1, and OPN were significantly lower in hBMSCs of lenti-POSTN group than lenti-control group at days 3 and 7 (*P* < 0.05, Figures 5A–D). Western blotting analysis revealed lower RUNX2 and COL1A1 protein levels in hBMSCs of the lenti-POSTN group than lenti-control group (Figures 5E–G), which was consistent with the results of qPCR.

### ALP Activity and Calcium Nodule Formation Were Attenuated After POSTN Knockdown

Alkaline phosphatase activity is an early stage marker of osteogenic differentiation of MSCs. We evaluated the effects of POSTN knockdown on ALP activity during osteogenic differentiation. The results revealed that lower ALP activity in POSTN knockdown hBMSCs than in the lenti-control group at days 7 during osteogenic differentiation (*P* < 0.05, Figure 5I), and ALP staining suggested the similar results (Figure 5H). Calcium nodule formation was detected by ARS staining and the quantification of stained areas were performed by measuring the absorbance at 570 nm. Fewer calcium deposits were observed in the lenti-POSTN group than lenti-control group at days 14 (Figure 5J). The results of the quantification analysis indicated similar results (Figure 5K).

### POSTN Knockdown Increased SOST Expression and Inhibited the Wnt/β-Catenin Signaling Pathway

**FIGURE 6:**
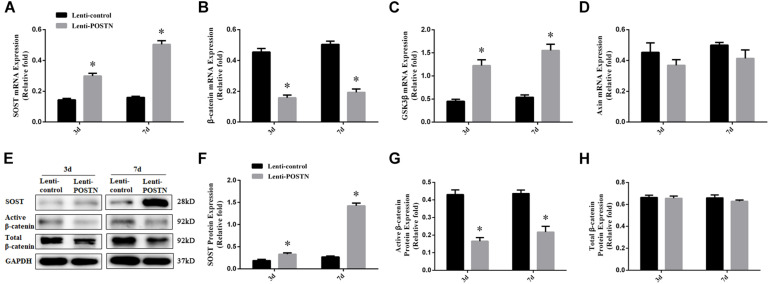
POSTN knockdown upregulated SOST expression during osteogenesis and downregulated the Wnt/β-catenin signaling pathway during osteogenic differentiation of hBMSCs in patients with SONFH. **(A)** The mRNA levels of SOST were detected by qPCR on days 3 and 7 after osteogenic induction of hBMSCs. **(B)** The mRNA levels of β-catenin. **(C)** The mRNA levels of GSK3β. **(D)** The mRNA levels of Axin. **(E–H)** The protein levels of SOST, Active β-catenin, and Total β-catenin were detected by western blotting analysis on days 3 and 7 after osteogenic induction of hBMSCs. All experimental data are given as mean values ± SD and were confirmed by three independent experiments (*n* = 3) with cells from three different donors. ^∗^*P* < 0.05 versus the lenti-control group.

To assess the effects of POSTN knockdown on SOST expression, and to further confirm whether the effects of POSTN on osteogenic differentiation of hBMSCs in patients with SONFH is associated with Wnt/β-catenin signaling, the expression levels of SOST and β-catenin were detected by qPCR and western blotting at days 3 and 7 during osteogenesis of hBMSCs. The mRNA expression levels of GSK3β and Axin were also measured by qPCR. The results of the qPCR and western blotting showed higher expression levels of SOST (Figures 6A,E,F), while lower expression levels of β-catenin in POSTN knockdown hBMSCs than in lenti-control group during osteogenic differentiation (Figures 6B,E,G). Compared with the lenti-control group, the mRNA expression of GSK3β was upregulated in POSTN knockdown hBMSCs (Figure 6C); however, the mRNA expression of Axin and the protein expression of Total β-catenin did not change (Figures 6D,E,H).

## Discussion

The pathogenesis of steroid-induced osteonecrosis of femoral head (SONFH) has not been fully elucidated, and is considered to be the result of multiple complex pathological mechanisms. Among them, the decreased activity and abnormal differentiation of bone marrow mesenchymal stem cells (BMSCs) may be an important mechanism for the occurrence and development of SONFH (Hernigou and Beaujean, 1997; Houdek et al., 2016; Han et al., 2019). Cui et al. (1997) found that pluripotential bone-marrow cells from mouse bone-marrow stroma treated by glucocorticoids (GCs) tended to differentiate into adipocytes rather than osteoblasts *in vitro*. BMSCs isolated from SONFH patients displayed a decreased ability to differentiate into bone but preferentially differentiate into adipose tissue or cartilage, due to the long-term exposure to GCs (Houdek et al., 2016). In another study, Hao et al. (2016) reported that miR-708 can inhibit the osteogenic differentiation of hBMSCs by targeting SMAD3, thereby promote the occurrence and development of SONFH. Besides, GCs inhibit the osteogenic differentiation of MSCs *in vitro* and bone formation *in vivo* by down-regulating the expression of osteogenesis-related genes such as RUNX2 and ALP (Han et al., 2019). Therefore, it is crucial for the treatment of SONFH by promoting osteoblastogenesis and inhibiting adipogenesis of MSCs to promote bone remodeling and restore osteo-adipogenic balance.

In the skeleton, POSTN is preferentially expressed by periosteal osteoblasts and osteocytes in response to mechanical stimuli and parathyroid hormone (PTH), and plays a key role in regulating osteoblast function and promoting bone formation (Bonnet et al., 2016). Osteoblast differentiation *in vitro* and bone formation *in vivo* can be promoted and accelerated by injecting an adenovirus overexpressing POSTN into the marrow cavity of rats (Zhu et al., 2009). Conversely, after treatment of mouse MC3T3-E1 cells with anti-POSTN antibody, the expression of osteoblast-specific-differentiation markers, such as RUNX2, collagen type I, osteocalcin, osteopontin and ALP, as well as calcium deposition was markedly decreased during the process of osteoblast differentiation *in vitro* (Litvin et al., 2004). Besides, altered bone material properties and increased damage accumulation with delayed intracortical remodeling and decreased callus formation were observed in POSTN-deficient mice (Bonnet et al., 2013). In another study, the results of increased SOST expression and not to be suppressed by axial compression in POSTN^–/–^ mice indicated that POSTN is required for SOST inhibition and play an important role in maintaining bone mass and trabecular microstructure in response to loading (Bonnet et al., 2009).

To our best knowledge, the present study is the first to investigate the effects of POSTN on regulating SOST expression and osteogenic differentiation of hBMSCs in patients with SONFH. The results of qPCR and western blotting analysis revealed that the endogenous expression of POSTN and SOST in hBMSCs of SONFH patients was upregulated. Western blotting analysis indicated that the expression of POSTN was increased gradually while SOST was decreased gradually during the osteogenic differentiation of hBMSCs in Control Group. However, the expression of POSTN and SOST showed opposite trends during the osteogenic differentiation of hBMSCs in SONFH Group, which may be one of the reasons for the attenuated osteogenic differentiation ability of hBMSCs caused by glucocorticoid excess. Besides, this phenomenon is possibly explained by an increased level of SOST expression in hBMSCs as a consequence of glucocorticoid treatment. Conversely, the enhanced POSTN expression in hBMSCs could antagonize the increasing SOST expression during the osteogenic differentiation of hBMSCs in patients with SONFH. Therefore, to further study the exact role of POSTN during osteogenic differentiation of hBMSCs in patients with SONFH, we used a POSTN knockdown strategy to study its effects on the osteogenic differentiation ability of hBMSCs. RUNX2, a major transcription factor of osteoblast differentiation (Komori et al., 1997), was significantly reduced in expression at days 3 and 7 during osteogenic differentiation after POSTN knockdown, as detected by qPCR and western blotting. Similar results were observed in the expression patterns of other specific osteogenic markers, such as OSX, COL1A1, and OPN. ALP activity and calcium deposits are recognized as early and late markers of osteoblast differentiation, respectively (Chen et al., 2015). We found that POSTN knockdown attenuated ALP activity and reduced mineralization during osteogenic differentiation of hBMSCs. Meanwhile, POSTN knockdown did not affect cell proliferation of hBMSCs in patients with SONFH. Accordingly, these results suggest that POSTN knockdown suppressed the osteogenic differentiation of hBMSCs in patients with SONFH *in vitro*.

Wnt/β-catenin signaling pathway, mediated by β-catenin, plays a crucial role in regulating osteogenic differentiation of MSCs and bone formation (Park et al., 2015). In the absence of Wnt ligand, β-catenin in the cytoplasm is degraded by a multiprotein destruction complex, including GSK3β, adenomatous polyposis (APC), and tumor suppressors Axin. Beta-catenin is brought to GSK-3β and casein kinase 1 (CK1) by APC and Axin for phosphorylation. Subsequently, phosphorylated β-catenin is targeted for polyubiquitination and proteosomal degradation to prevent its accumulation in the cytoplasm. On the other hand, under the influence of the Wnt ligand, Disheveled together with Axin-GSK3β is recruited by frizzled and LRP-5/6 co-receptors to the plasma membrane. The formation of the β-catenin complex is then inhibited and β-catenin levels in the cytoplasm increase, resulting in β-catenin translocation to the nuclear and transcriptional activation of target genes (Rudnicki and Williams, 2015; Nusse and Clevers, 2017). Moreover, the increased expression levels of non-phosphorylated (active) β-catenin are important indicators for activating the Wnt/β-catenin pathway, while GSK-3β is a crucial negative regulator of the Wnt/β-catenin pathway (Li et al., 2017). Therefore, the inhibition of GSK-3β activity is crucial for activating the Wnt/β-catenin pathway to promote osteoblast differentiation of BMSCs and bone formation (Martino et al., 2016). In our current study, POSTN knockdown increased GSK-3β expression and reduced the expression levels of β-catenin during osteogenic differentiation of BMSCs in patients with SONFH, as determined by qPCR and western blotting analysis, which was consistent with the previous study. Besides, SOST acted as an inhibitor of Wnt signaling was significantly increased in expression at days 3 and 7 during BMSC osteogenesis after POSTN knockdown, as detected by qPCR and western blotting. These findings indicate that POSTN knockdown upregulates SOST expression during osteogenesis and inhibits osteogenic differentiation of BMSCs in patients with SONFH via inactivation of the Wnt/β-catenin signaling.

There have been many studies to investigate the effect of POSTN or SOST on bone formation and bone remodeling through gene knockout animal models of POSTN or SOST. However, this study is the first to elucidate the impact of POSTN knockdown on SOST expression and osteogenic differentiation of hBMSCs in patients with SONFH. Inevitably, there are still several limitations to our study. Firstly, our findings were obtained using two-dimensional (2D) monolayer cell cultures, which do not adequately mimic the *in vivo* microenvironment exposed to GCs and present important limitations, such as reduced cell growth rates, cellular senescence, and low differentiation efficiency of MSCs (Wang et al., 2009; Tietze et al., 2019). In recent years, three-dimensional (3D) culture systems have been developed and exhibit multiple advantages, such as mimicking the native 3D environment, facilitating the interaction and communication between cells, increasing extracellular matrix (ECM) secretion, and potentiating osteogenic differentiation of MSCs (Zhang et al., 2015; Park et al., 2018). Hence, further experiments should be conducted using 3D culture systems to verify these findings in the future. Secondly, it is possible that the effects of POSTN knockdown on SOST expression and osteogenic differentiation of hBMSCs *in vitro* may not be exactly consistent with corresponding changes *in vivo*, so the function of POSTN and SOST and possible heterogeneity between *in vitro* and *in vivo* experiments should be further investigated *in vivo* for greater biological relevance. Lastly, the effect of only POSTN knockdown but not POSTN overexpression on osteogenic differentiation of hBMSCs in patients with SONFH was determined in the present study. Furthermore, the exact mechanisms underlying the inactivation of the Wnt/β-catenin signaling pathway by POSTN knockdown, especially in terms of β-catenin nuclear translocation, remain to be clarified. Whether there are other signaling pathways potentially involved in the alteration of BMSC osteogenic differentiation in patients with SONFH caused by the knockdown or overexpression of POSTN needs to be investigated in the future. Besides, due to the insufficient clinical samples in this study, whether the expression levels of POSTN and SOST are closely associated with the staging and progression of SONFH still requires large sample data of experimental and clinical research for statistical analysis.

## Conclusion

In summary, our results suggest that the POSTN knockdown of hBMSCs in patients with SONFH facilitates SOST expression during osteogenesis and inhibits osteogenic differentiation of hBMSCs, partially via inactivation of the Wnt/β-catenin signaling pathway. Hence, promoting POSTN expression or inhibiting SOST expression in hBMSCs of SONFH patients may provide a promising stem cell-based strategy for tissue engineering and regenerative medicine including bone regeneration.

## Data Availability Statement

The raw data supporting the findings of this study will be made available by the authors upon reasonable request.

## Ethics Statement

The studies involving human participants were reviewed and approved by Ethics Committee of Tongji Medical College, Huazhong University of Science and Technology. The patients/participants provided their written informed consent to participate in this study.

## Author Contributions

WX and LH conceived and designed the study. LH and SG contributed to carry out the experiments. SL and BW contributed to data analysis. LH wrote the manuscript and designed the figures. GC and TG contributed to the writing and editing of the manuscript. WX and RW supervised the research. All the authors and participants reviewed the manuscript and approved the final manuscript.

## Conflict of Interest

The authors declare that the research was conducted in the absence of any commercial or financial relationships that could be construed as a potential conflict of interest.
